# Reactivation of a Transplant Recipient's Inherited Human Herpesvirus 6 and Implications to the Graft

**DOI:** 10.1093/infdis/jiae268

**Published:** 2024-05-20

**Authors:** Leo Hannolainen, Lari Pyöriä, Diogo Pratas, Jouko Lohi, Sandra Skuja, Santa Rasa-Dzelzkaleja, Modra Murovska, Klaus Hedman, Timo Jahnukainen, Maria Fernanda Perdomo

**Affiliations:** Department of Virology, University of Helsinki and Helsinki University Hospital, Finland; Department of Virology, University of Helsinki and Helsinki University Hospital, Finland; Department of Virology, University of Helsinki and Helsinki University Hospital, Finland; Department of Electronics, Telecommunications and Informatics, Institute of Electronics and Informatics Engineering of Aveiro, and Intelligent Systems Associate Laboratory, University of Aveiro, Portugal; Department of Pathology, University of Helsinki and Helsinki University Hospital, Finland; Joint Laboratory of Electron Microscopy, Institute of Anatomy and Anthropology; Institute of Microbiology and Virology, Rīga Stradiņš University, Riga, Latvia; Institute of Microbiology and Virology, Rīga Stradiņš University, Riga, Latvia; Department of Virology, University of Helsinki and Helsinki University Hospital, Finland; Department of Pediatric Nephrology and Transplantation, Children's Hospital and Helsinki University Hospital, Finland; Department of Virology, University of Helsinki and Helsinki University Hospital, Finland

**Keywords:** virome, inherited chromosomally integrated human herpesvirus 6, host immune profiling, reactivation, transplantation

## Abstract

**Background:**

The implications of inherited chromosomally integrated human herpesvirus 6 (iciHHV-6) in solid organ transplantation remain uncertain. Although this trait has been linked to unfavorable clinical outcomes, an association between viral reactivation and complications has only been conclusively established in a few cases.

**Methods:**

We used hybrid capture sequencing for in-depth analysis of the viral sequences reconstructed from sequential liver biopsies. Moreover, we investigated viral replication through in situ hybridization (*U38–U94* genes), reverse transcriptase polymerase chain reaction (*U89/U90* genes), immunohistochemistry, and immunofluorescence. We also performed whole transcriptome sequencing to profile the host immune response.

**Results:**

We report a case of reactivation of a recipient’s iciHHV-6B and subsequent infection of the graft. Using a novel approach integrating the analysis of viral and mitochondrial DNAs, we located the iciHHV-6B intragraft. We demonstrated active replication via the emergence of viral minor variants, in addition to positive viral messenger RNAs and antigen stainings in tissue sections. Furthermore, we detected significant upregulation of antiviral immune responses, arguing against immunotolerance.

**Conclusions:**

Our analysis underscores the potential pathological impact of iciHHV-6B, emphasizing the need for monitoring reactivation in transplant recipients. Most crucially, it highlights the critical role that the host's virome can play in shaping the outcome of transplantation.

**(See the Editorial Commentary by Heldman et al. on pages e263–6.)**

Human herpesvirus 6 (HHV-6) is a ubiquitous pathogen known to establish latency by integrating into the subtelomeric regions of human chromosomes [1]. If the integration takes place within germ cells, the HHV-6 DNA can be transmitted vertically through Mendelian inheritance, resulting in a condition referred to as inherited chromosomally integrated HHV-6 (iciHHV-6). This phenomenon is observed in approximately 1% of the population [2].

While HHV-6 as such causes substantial morbidity and mortality in transplant recipients [3], especially in those receiving allogeneic hematopoietic stem cells [4], there is scarcity of data on the clinical impact of iciHHV-6 in transplantation. Indeed, given its low frequency, the existing knowledge is mainly based on case reports, which most often fail to provide concrete evidence of iciHHV-6 reactivation and of the subsequent effects on the graft or the recipient. However, higher rates of graft-versus-host disease [5], bacterial infections, and allograft rejections have been reported among transplant recipients carrying iciHHV-6 [6], suggesting that this hereditary trait bears an increased risk for complications.

Among solid organ transplantations, only 3 case reports have comprehensively investigated clinical outcomes after reactivation of iciHHV-6 carried with the implant [7–9]. In this study, for the first time, we demonstrated the reactivation of a host's inherited virus, iciHHV-6B, providing several layers of evidence of reactivation and infection of the liver graft. In support of this, we introduced a unique approach that integrates the analysis of viral and mitochondrial DNAs for spatial resolution of the viral infection. Moreover, using whole transcriptome analysis, we showed the contemporary occurrence of substantial antiviral immune responses in the graft, arguing against immunotolerance toward this inherited virus.

Our study highlights the pathogenicity of iciHHV-6 and the clinical importance of excluding reactivation, beyond the high baseline copies inherent to this trait [10]. Crucially, our work redefines the transplantation landscape, emphasizing that the outcome is not solely shaped by the graft but hinges on the complex dynamics of the host's virome [11].

## CASE REPORT

A pediatric patient (recipient [R]) with biliary atresia received a liver transplant (LTx) due to sustained cholestasis and portal hypertension, despite a Kasai operation at the age of 1 month. The transplant involved segments 2 and 3 of a deceased donor liver (human cytomegalovirus [HCMV] donor positive/recipient negative; Epstein-Barr virus [EBV] donor not measured/recipient negative; HHV-6 donor not measured/recipient positive). The plasma alanine aminotransferase (ALT) decreased from 209 U/L to 87 U/L and the bilirubin from 464 μmol/L to 48 μmol/L within the first 2 posttransplant weeks, but never reached reference values. Ammonia and factor V levels normalized rapidly after LTx, indicating good graft function. Repeated ultrasonography showed normal flow in the hepatic artery and portal vein.

At the time of LTx, 4.6 million copies/mL of HHV-6 DNA were detected in whole blood, with rising loads during the following 2 weeks. Intravenous ganciclovir was initiated due to a concurrent ALT increase in plasma from 87 to 116 U/L and emerging clinical symptoms including fever and vomiting. One day later, a blood culture revealed *Enterococcus raffinosum*, and vancomycin was added to a meropenem course initiated earlier. Antiviral therapy was continued with oral valganciclovir. Additionally, the patient developed HCMV viremia (peak 3100 IU/mL 2 weeks later), prompting discontinuation of azathioprine and adjustment of tacrolimus levels to 5–7 ng/mL. The patient's clinical condition improved but because the ALT and bilirubin did not normalize, a graft biopsy was performed on day 24 post-LTx.

**Figure 1.**
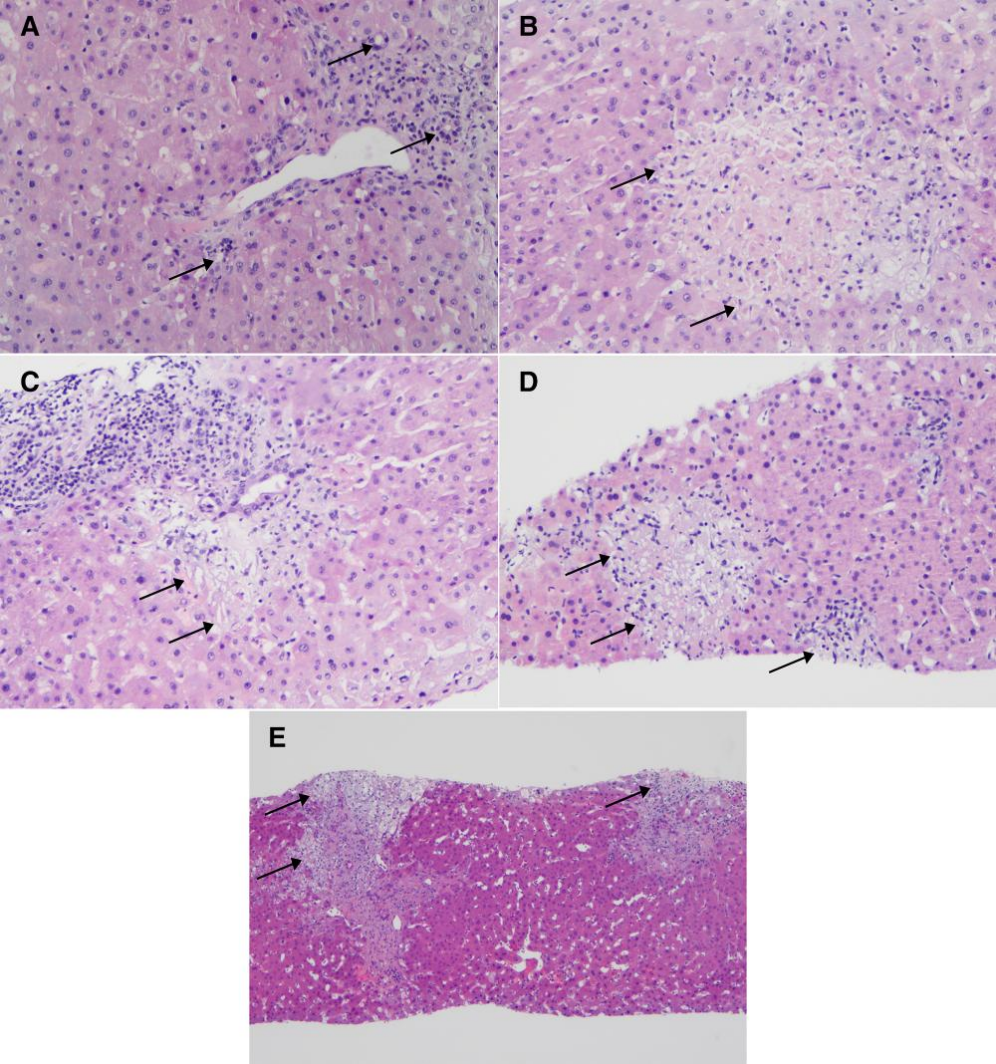
Histology of the liver biopsies. Biopsy taken on day 24 post–liver transplant (LTx) showing with arrows, at 100× original magnification, mild portal lymphocytic infiltrate and mild cholangitis (*A*), necrotic foci in the periportal lobular liver parenchyma (*B*), and mild portal lymphocytic infiltrate, mild cholangitis, and a periportal necrotic focus (*C*). *D*, Biopsy from day 58 post-LTx showing mild cholestasis, persisting mild portal lymphocytic infiltrate, mild cholangitis, and a periportal necrotic focus (100× original magnification). *E*, Biopsy taken on day 100 post-LTx showing enlargement and fibrosis of portal tracts, persisting mild portal lymphocytic infiltrate, and periportal necrotic foci (40× original magnification).

The biopsy showed no signs of allograft rejection. Instead, mild cholangitis, portal edema, and areas of micronecrosis in the liver parenchyma (1%–2%) were found, suggestive of viral infection (Figure 1*A*–*C*). HCMV and herpes simplex virus antigen stainings were negative.

A magnetic resonance cholangiopancreatography (MRCP) with gadoxetate disodium showed biliary leakage in the bile-gut anastomosis. A biliary-duodenal anastomosis reconstruction was performed 4 weeks after LTx, and a percutaneous transhepatic (PTC) drain was inserted. Due to increasing ALT and bilirubin plasma levels, 2 other consecutive biopsies were performed at 2 and 3 months post-LTx, in which no signs of allograft rejection were found, but instead, micronecrotic lesions covering up to 2% of the parenchyma, and persistent mild cholangitis (Figure 1*D*). At 100 days post-LTx, the portal tracts were enlarged and fibrotic (Figure 1*E*). In MRCP, the biliary tract was universally dilated and percutaneous transhepatic cholangiography in segments 2 and 3 showed narrowed lumina. There was no stenosis in the bilio-duodenal anastomosis. A new PTC drain was inserted. The biliary tract dilation disappeared, yet the bacterial cholangitis recurred and cholestasis persisted. The patient was listed for retransplantation approximately 3 months after the first LTx. The explanted graft showed severe portal fibrosis, severe cholestasis, and loss of small portal bile ducts. A large central biliary duct was destroyed.

As the exact cause of the chronic cholangitis and liver lesions was unclear, HHV-6 was considered a potential contributor given the heavy viremia. During the second LTx, basiliximab induction therapy was omitted, and intravenous ganciclovir was initiated immediately after surgery. The HHV-6 levels decreased, and the liver function improved.

A protocol graft biopsy 3 months after the second transplant showed no significant abnormalities.

## METHODS

The study was approved by the Ethics Committee of the Helsinki and Uusimaa Health District (protocols 939/2018 and HUS/462/2021). Informed consent was obtained from the parents before the study.

Materials available for analysis included formalin-fixed, paraffin-embedded (FFPE) tissue sections (5 μm) of the explant and implant collected at the time of transplantation (baseline) as well as follow-up biopsies taken at 1, 2, 3, and 6 months posttransplantation. Blood samples were collected according to the routine follow-up protocol.

In addition, a blood sample was collected from the parents to verify an inherited chromosomal integration of HHV-6.

### DNA Extraction

The DNA from FFPE biopsies was extracted using the QIAamp DNA FFPE Advanced kit (Qiagen), according to the manufacturer’s protocol, and eluted in 100 μL of ATE buffer. Negative control samples (phosphate-buffered saline [PBS]) were included.

Blood samples from the parents were extracted using the QIAmp DNA blood mini kit (Qiagen) following the manufacturer's instructions and eluted in 60 μL of AE buffer. Nuclease-free water was processed as a negative control.

### Virus DNA Detection by Quantitative Polymerase Chain Reaction

Quantitative polymerase chain reaction (qPCR) was performed to type and quantify all the human herpesviruses (herpes simplex virus [HSV] 1, HSV-2, varicella zoster virus, EBV, HCMV, HHV-6A, HHV-6B, HHV-7, and Kaposi sarcoma–associated herpesvirus) as described by Pyöriä et al [12]. In addition, qPCRs were performed for Torque teno virus and human parvovirus B19 as described previously [13, 14], to confirm the results obtained by next-generation sequencing (NGS). RNaseP qPCR was also performed for quantification and normalization of the viral copies per million cells as described [13].

### Hybrid Capture Sequencing

Hybrid capture sequencing was performed on the liver biopsies from the explant and implant at months 0, 1, 2, and 3.

The DNA was mechanically fragmented with Covaris E220 with a target length of 200 nucleotides. Subsequently, the libraries were prepared with the KAPA HyperPrep kit (Roche) using unique Dual Index Adapters (Roche). Targeted enrichment of the viral DNAs was performed using a custom panel of biotinylated RNA probes (Arbor Biosciences) as described elsewhere [14] and the sequencing performed on NovaSeq 6000 (1 lane, S4, PE151 kit; Illumina).

The data analysis was performed using TRACESPipe as described previously [15]. Further details on the targeted enrichment and pipeline analysis are shown in Supplementary Text 1.

### Variant Calling

Minor variants were called from the BAM files created by TRACESPipe using IVAR [16], after masking of repeat regions. The thresholds used were a *P* value of <.05 and an alternative nucleotide frequency >3%.

The minor-variant position density was calculated by the number of positions divided by genome length and position-wise Shannon entropy by function H = −∑pi × log2(pi) for every site.

### Reverse-Transcription PCR

The RNA was extracted from FFPE sections of the explant (timepoint 0) and implant (timepoints 0 and 6 months), using the PureLink FFPE Total RNA Isolation Kit (Invitrogen) according to the manufacturer's protocol. Complementary DNA (cDNA) was synthesized by reverse transcription using the RevertAid First Strand cDNA Synthesis Kit (Thermo Scientific). To assure the quality of cDNA, PCR was carried out to detect the β-globin gene according to Vandamme et al [17]. The HHV-6A/B U89/90 immediate-early gene expression was detected according to Van den Bosch et al [18]. Electrophoretic analysis was done to separate and identify the PCR-amplified fragments.

### Immunohistochemistry and Immunofluorescence

The tissue sections were deparaffinized in xylene and dehydrated in a series of graded ethanol. Endogenous peroxidase activity was blocked using 3% hydrogen peroxide in methanol. For antigen retrieval, the sections were boiled in citrate buffer (pH = 6) and then incubated with the primary mouse monoclonal antibody HHV-6 (20) raised against viral lysate, using a 1:200 dilution (sc-57804, Santa Cruz Biotechnology).

For routine immunohistochemistry, amplification of the primary antibody and visualization of reaction products were performed by applying the HiDef DetectionTM HRP Polymer system (Cell Marque) and the 3,3′ diaminobenzidine (DAB) tetrahydrochloride kit (DAB + chromogen and DAB + substrate buffer, Cell Marque). The sections were counterstained with Mayer's hematoxylin, washed with tap water, dehydrated, cleared, and mounted in polystyrene. Negative controls consisting of PBS instead of the primary antibody were included. Positive control samples included tissues from encephalopathy and arthritis cases positive for HHV-6 by nested PCR and antibody-based assays.

For immunofluorescence, after immunostaining with the primary antibody, the sections were washed with PBS, followed by 1:300 dilution of fluorescent secondary antibody (goat anti-mouse immunoglobulin G [H + L], Alexa Fluor 488 conjugated from Thermo Fisher Scientific). The sections were counterstained with 4′,6-diamidino-2-phenylindole (DAPI) and embedded in Prolong Gold and DAPI for visualization of nuclei.

### RNA In Situ Hybridization

FFPE tissue sections of the explant and implant (months 1–6) were mounted into SuperFrost slides, and messenger RNA (mRNA) in situ hybridization of the U38 and U94 genes of HHV-6 was performed using the RNAscope 2.5 HD Assay-RED (ACD) according to the manufacturer´s protocol. The human PPIB mRNA probe and bacterial DapB probe were included as positive and negative technical controls, respectively.

### Transcriptome Analyses

RNA from graft tissue sections (timepoints 0, 2, and 6) was extracted with the RNAeasy FFPE extraction kit (Qiagen) after deparaffinization with xylene, according to the manufacturer´s protocol. The extracts were eluted in 30 μL of RNAse-free water.

The library preparation, RNAseq, and bioinformatic analysis were performed by Novogene. The transcriptome reconstruction was performed using Trinity (comprising modules Inchworm, Chrysalis, and Butterfly) [19].

## RESULTS

We investigated the role of iciHHV-6 in the clinical course of a pediatric liver recipient with no signs of allograft rejection, instead with persistent cholangitis and areas of micronecrosis in the liver parenchyma, suggestive of viral infection (Figure 1).

Before transplantation, iciHHV-6 had been suspected based on the finding of 4.6 million copies/mL of HHV-6 DNA in whole blood and a virus/white blood cell (WBC) ratio of 1.5. We later confirmed this diagnosis through the finding of similar loads in 1 of the parents (virus/WBC ratio 1.1) and shared identical sequences of iciHHV-6B.

**Figure 2.**
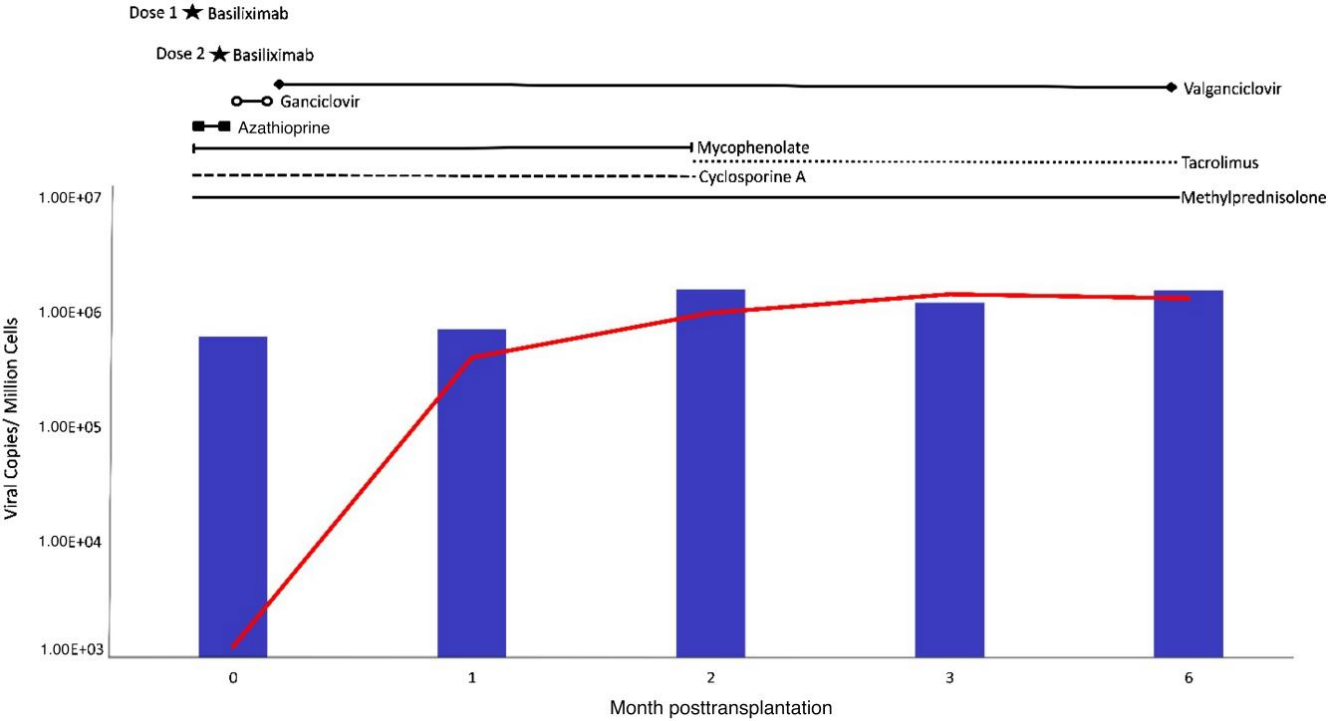
Copies of human herpesvirus 6B (HHV-6B) DNA in blood and liver. Loads of HHV-6B quantified by quantitative polymerase chain reaction in the patient’s blood (bars, copies/million leukocytes) and in liver biopsies (line, copies/million cells) collected at transplantation (time 0) and in consecutive months. On the top is the treatment course.

Shortly after transplantation, the HHV-6 loads increased, reaching a peak at 2 weeks of 39 million copies/mL. The viremia decreased to baseline after initiation of intravenous ganciclovir, followed by maintenance with oral valganciclovir, during which the copies in blood fluctuated, remaining elevated on average at 11.6 million copies/mL (Figure 2).

### The DNA Virome's Landscape Within the Explant and Implant

To investigate the implications of iciHHV-6, we tested biopsies of the liver implant collected at baseline (pre-perfusion, time 0) and at 1, 2, 3, and 6 months, as well as of the explant.

We performed a multiplex qPCR to detect and quantify all 9 human herpesviruses, as well as a customized hybrid capture protocol that targets 41 human DNA viruses followed by NGS. The prevalence of other viruses (than HHV-6B) detected using these methods is presented in Supplementary Table 1.

At the time of transplantation (time 0), both the explant and the implant were positive for HHV-6B DNA, albeit the latter at 4000-fold lower copies (1230 copies/million cells in the implant vs 4 890 000 copies/million cells in the explant) (Figure 2).

Increasing levels of HHV-6B DNA were detected in the follow-up liver biopsies (months 1–6), with a maximum of 1.5 million copies/million cells detected at month 3. The biopsies were negative for other herpesviruses, except those collected at month 6, in which low copies of EBV, HCMV, and HHV-7 were detected (200, 6, and 100 copies/million cells, respectively; Supplementary Table 1).

### Sequencing and Localization of iciHHV-6B Intragraft

To determine a potential reactivation of HHV-6B and to distinguish whether it originated from the graft’s HHV-6B or the patient´s iciHHV-6B, we performed hybrid capture sequencing.

**Figure 3.**
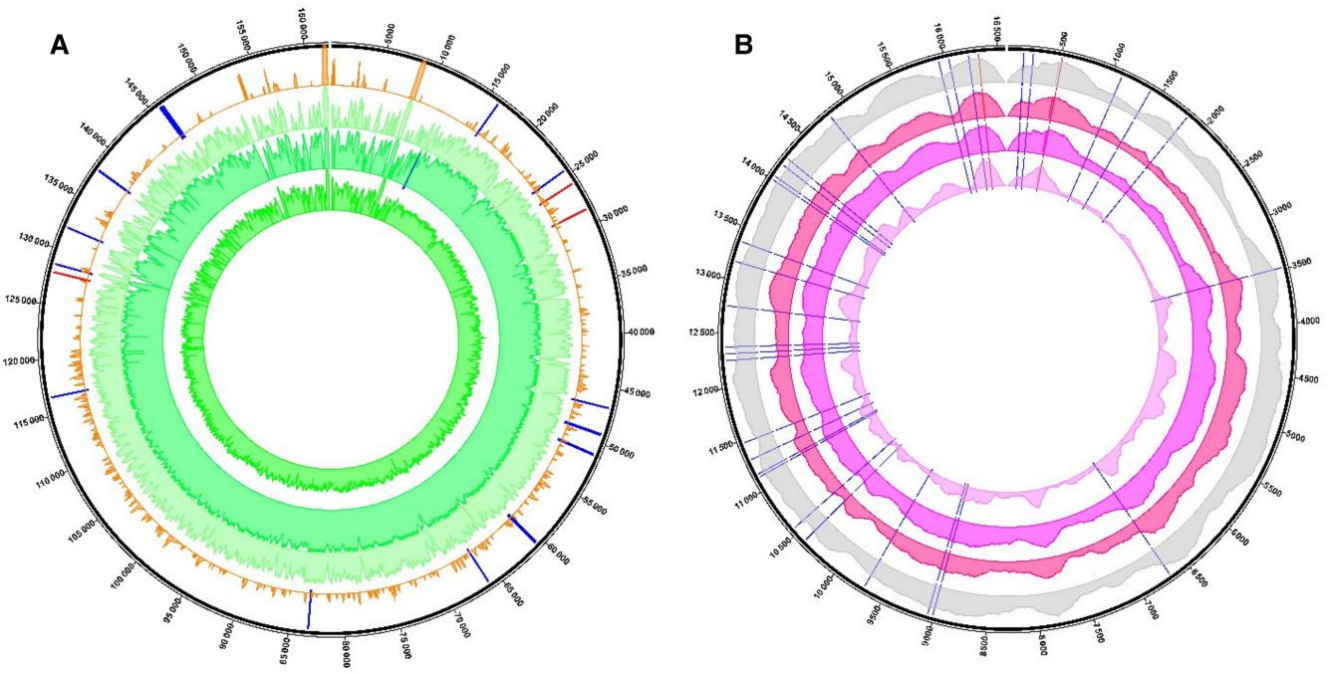
Genomic analysis of human herpesvirus 6B (HHV-6B) and mitochondrial DNA from liver biopsies. From outer to inner, represented are the genomic analysis of the sequences of HHV-6B (*A*) and mitochondrial DNA (mtDNA) (*B*) reconstructed from the explant (external solid circles) and the implant (orange or gray, respectively) at baseline. The following consecutive circles represent, in green (*A*) or in pink (*B*), HHV-6B or mtDNA sequences, respectively, recovered from the liver biopsies collected at 1, 2, and 3 months post–liver transplant (from outer to inner). The lines represent differences between genomes at the specified positions, with single-nucleotide polymorphisms in blue, or insertions and deletions in red. The sequence coverage is shown logarithmically in (*A*) but not in (*B*). The respective coverages of the explant and implant at baseline were for the inherited chromosomally integrated HHV-6 50X and 2X (64 426 and 2769 reads), and for the mtDNA 22X and 300X (3074 and 27 112 reads mapped).

From the biopsies collected at baseline, we reconstructed 97% (158 kb/162 kb) of the iciHHV-6B genome of the explant (50X coverage) and 41% (68 kb/162 kb) of the HHV-6B of the implant (2X). The sequences differed in 125 positions, of which 113 were single-nucleotide polymorphisms (SNPs) and 12 were insertions and deletions (INDELs). These changes translated into 38 synonymous and 31 nonsynonymous amino acid substitutions (Figure 3*A*).

In the liver biopsies taken 1, 2, and 3 months post-LTx, the HHV-6B sequences recovered were essentially identical to the iciHHV-6B (median coverage 40X; Figure 3*A*).

To resolve whether the reconstructed iciHHV-6B sequences were reflecting the parenchyma, as opposed to the recipient’s own circulating cells, we analyzed from these same sequencing data the mitochondrial DNA (mtDNA). We hypothesized that if the iciHHV-6B detected in the liver biopsies was mirroring the patient’s cells, a composite mtDNA genetic profile (ie, a mixed pattern of mtDNA from donor and recipient) would be recovered. Instead, the mtDNA sequences (median coverage 215X for months 1, 2, and 3) matched exclusively that of the donor, suggesting that the iciHHV-6B was indeed intragraft (Figure 3*B*). The mtDNA genomes from the explant and implant corresponded to haplogroups R1b1 and K1c2, respectively, and differed in 35 positions, of which 32 were SNPs and 3 INDELs. These translated into 6 synonymous and 14 nonsynonymous mutations (Figure 3*B*).

Interestingly, the mtDNA genome of the recipient had 2 dominant mutations, A8860G and A15326G, located in the ATPase 6 of complex V and the cytochrome b of complex III respectively, which have been correlated with liver dysfunction in biliary atresia, the primary condition of this patient [20].

### Increase in the Number of Minor Variants Consistent With Reactivation

**Figure 4.**
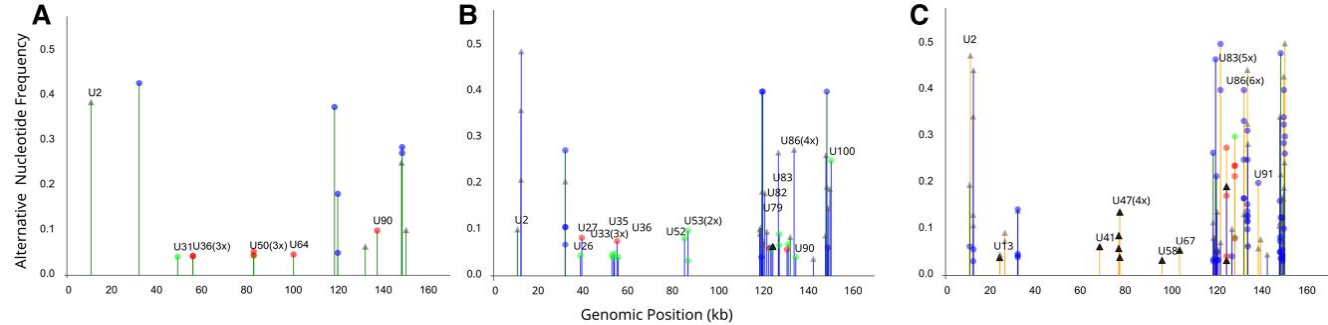
Minor-variant analysis of inherited chromosomally integrated human herpesvirus 6B across time. Represented are the minor-variant frequencies (y-axis) determined in the explant (*A*) and the graft at months 1 (*B*) and 2 (*C*) post-LTx, plotted against the reference genome NC_000898.1. The colors of the vertical lines indicate the time points when a particular minor variant was first observed: explant (green), 1 month (blue), and 2 months (orange). Circles represent single-nucleotide polymorphisms with synonymous mutations shown in green, nonsynonymous mutations in red, and mutations outside of coding sequences in blue. Gray triangles depict indels, whereas black triangles indicate indels leading to frameshift mutations within a gene. Genes are labeled above the vertical line and the numbers of minor-variant positions within the gene are in parentheses. The median sequencing depth was 46X.

We further analyzed the iciHHV-6B sequences to examine changes in genetic diversity over time. More specifically, we assessed the emergence of minor variants resulting from mutations occurring during replication. Across time points, we observed an increase in the number of minor variants, from 18 in the explant to 50 and 97 by months 1 and 2, respectively (Figure 4).

This rise in genetic diversity was also evident in the per-site Shannon index, which considers the frequencies of alternative nucleotides (Supplementary Figure 1). By month 3, the number of variants had decreased to 23, although this decline could be attributed to a lower sequencing depth.

Taken together, the increasing DNA copies determined in blood and liver post-LTx, as well as the sequencing data, were suggestive of reactivation of the recipient’s iciHHV-6B, followed by infection and replication in the graft.

### Reactivation of iciHHV-6 In Vivo

To confirm active replication, we investigated the expression of viral mRNAs by RT-PCR targeting the immediate-early gene *U89/90*, and by RNA in situ hybridization targeting the polymerase's early gene *U38* and the latent *U94* gene.

**Figure 5.**
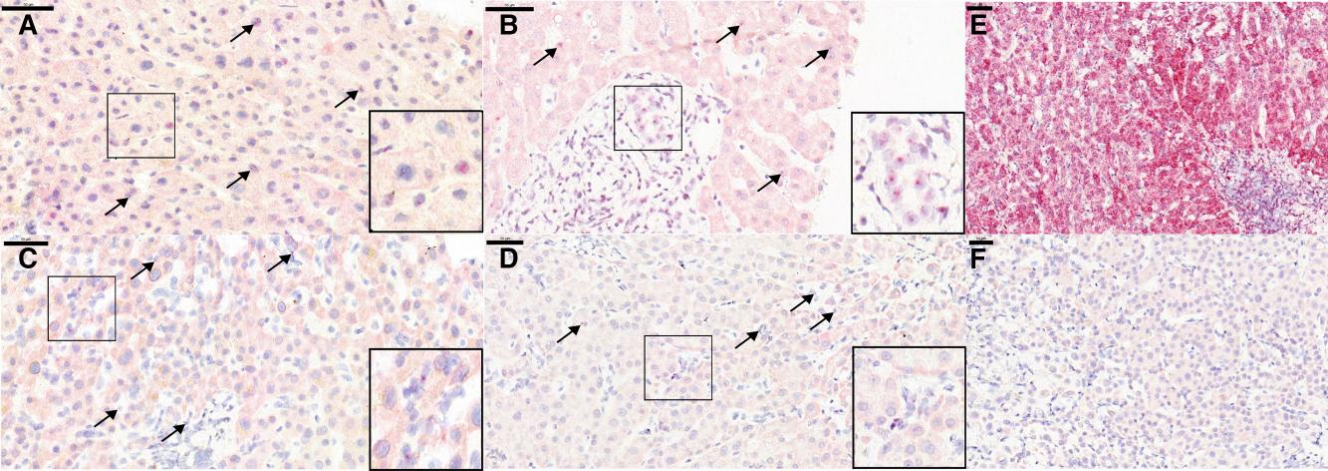
Detection of human herpesvirus 6 messenger RNA by RNAscope 2.5 HD Assay-RED in liver biopsies. Transcripts of the early U38 viral polymerase gene detected in the implant at 1 mo (*A*), 2 mo (*B*), 3 mo (*C*), and 6 mo (*D*) posttransplantation and counterstained with hematoxylin. Positive and negative technical controls of human PPIB probe (*E*) and bacterial DapB probe (*F*) were included. Representative positive probe signals are indicated with arrows and zoomed-in in the bottom right corner of each panel. Scale bar of 50 μm in the left upper corner. Images captured with CaseViewer 2.4. *A–D*, 20× magnification. *E* and *F*, 30× magnification.

By each method, viral transcription was detected in the graft (Figure 5 and Supplementary Figures 2 and 3), with heterogeneous mRNA expression in hepatocytes, biliary cells, and inflammatory cells determined by RNA in situ hybridization (Figure 5 and Supplementary Figure 3).

**Figure 6.**
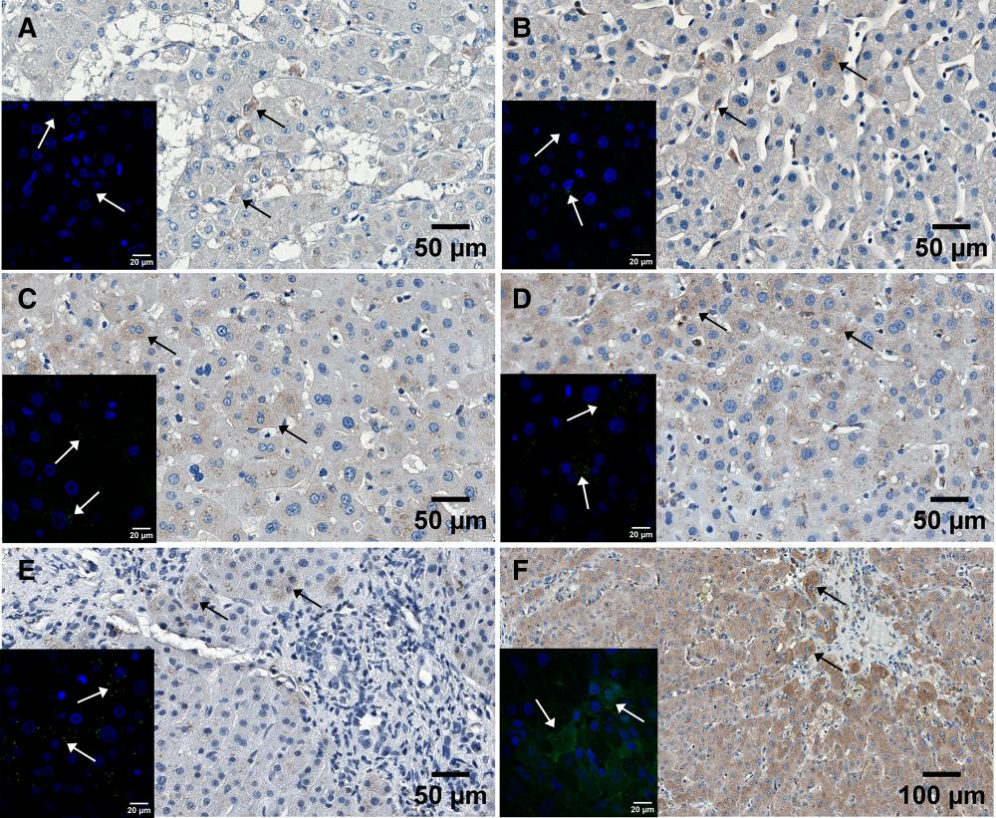
Detection of human herpesvirus 6 (HHV-6) antigens in liver biopsies. Positive staining (brown signal—3,3′ diaminobenzidine) of hepatocytes was detected by immunohistochemistry and counterstained with hematoxylin (nuclei—blue [DAPI]). Representative hepatocytes positive for HHV-6 antigens are indicated with black arrows in the explant (*A*) and the implant (*B*) at baseline and at months 1 (*C*), 2 (*D*), 3 (*E*), and 6 (*F*). The images were captured using bright field microscopy with original magnifications of 400× for *A–E* and 200× for *F*. Representative inserts in each microphotograph correspond to HHV-6 antigens (green) detected by immunofluorescence within hepatocytes (white arrows). Confocal microscopy (original magnification 1000×).

We also demonstrated HHV-6 protein expression in the liver biopsies by immunohistochemistry and immunofluorescence, using a HHV-6 monoclonal antibody against viral lysate. Analogously to RNAscope, the HHV-6 reactivity was observed primarily in the cytoplasm of hepatocytes, but also within the bile ducts and inflammatory cells. The most intensive antigen expression was detected at month 6 (Figure 6).

### Ongoing Antiviral Immune Responses Intragraft

Given the endogenous nature of iciHHV-6 and the potential immunotolerance against this virus, we performed whole transcriptome sequencing on the biopsies of the graft collected at baseline and at months 2 and 6 post-LTx, to profile the host immune response.

**Figure 7.**
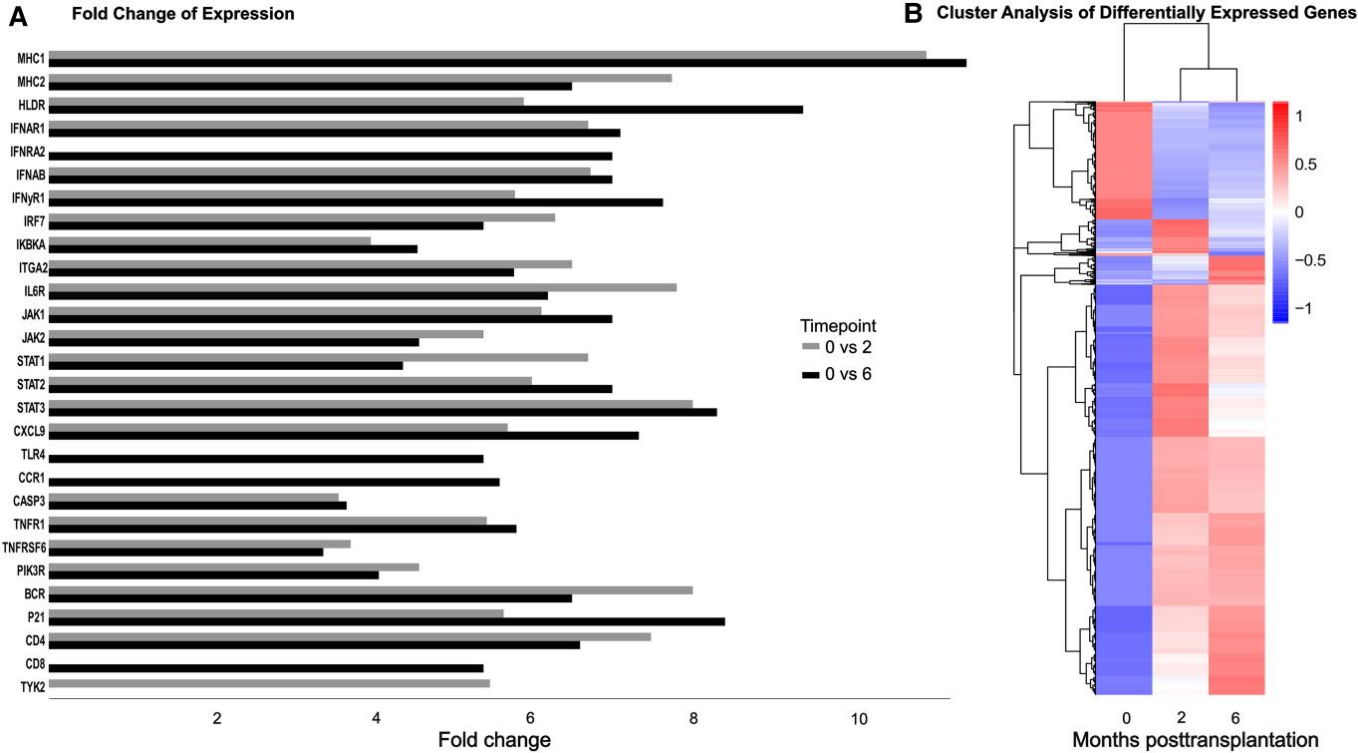
Differential gene expression by whole transcriptome sequencing. *A*, Selected immune response–related genes with the greatest fold change (*P* < .001) at month 2 (gray) and month 6 (black) in comparison to baseline. *B*, Cluster analysis of differentially expressed genes using H-cluster, K_means_cluster, and SOM_cluster. The x-axis represents months post–liver transplant, and the y-axis represents the corrected expression level value.

In total, we obtained 105 511 772 reads from the implant (timepoint 0), 83 481 888 from month 2, and 89 431 034 from month 6 post-LTx (∼26G/sample). Between 0 and 2 months, we found 14 026 differentially expressed genes, of which 77.3% were upregulated, and between 0 and 6 months 12 636 differentially expressed genes, of which 77.9% were upregulated (Figure 7*B* and Supplementary Figure 4*A* and 4*B*). Based on pathway analysis, most of the upregulated genes were classified into metabolism or signal transduction, followed by infectious diseases, with a total of 1513 genes assigned as differentially expressed (Supplementary Figure 4*C*). We identified a statistically significant upregulation (*P* < .001) of genes involved in antiviral immune responses.

Among the genes that were upregulated by at least 3-fold from baseline were histocompatibility molecules (*MHC1* increased fold expression of 11.40 from baseline to month 6), interferon pathway-related genes (*IFNAR-1* and *-2, IRF-7* and *-9, INFyR1*), cytokines (CXCL9, IL6R), and transcription factors (*STAT-1, -2,* and *-3* and *JAK-1* and *-2*) that are involved in the recruitment and activation of immune cells (Figure 7). Moreover, by month 6 post-LTx, we detected a 5.4-fold increase in the transcription of *CD8* genes, indicative of the expansion or activation of CD8^+^ T cells in the liver.

We also determined a 4.7-fold increase by month 2 in the expression of *CD112* (also known as *NECTIN2*), which mediates the entry of HHV-6 into hepatocytes [21].

## DISCUSSION

The significance of iciHHV-6 in transplantation has thus far remained uncertain, although increased rates of graft-versus-host disease [5], bacterial infections, and allograft rejections [6] have been observed in connection with this trait. However, only 3 case reports in solid organ transplantations have documented iciHHV-6 reactivations (2 of A type and 1 of B type) leading to allograft dysfunction [7–9]. In those cases, however, the iciHHV-6 originated from the donor, unlike in ours, which was inherent to the recipient.

In this study, we describe a case of an iciHHV-6B positive pediatric patient who underwent 2 liver transplants. While the most likely cause of graft loss and retransplantation was biliary leakage, the exact cause of the necrotic liver lesions and cholangitis was unclear. Because of the temporal relationship between the increase in HHV-6B copy numbers, the patient's clinical symptoms, and liver histology (persistent micronecrotic foci and cholangitis), iciHHV-6B was considered a potential contributor.

We provided multiple layers of evidence substantiating infection of the liver by the recipient's iciHHV-6B, bolstered by robust antiviral immune responses within the graft. Our findings are in line with those of Gravel et al [22] and Endo et al [23], who first demonstrated the reactivation and pathogenicity of iciHHV-6 in vivo, albeit outside of the context of transplantation.

The impact of the iciHHV-6B reactivation on the outcome of this transplantation was possibly bipartite, not only as a direct consequence of liver infection but also by recruitment of immune cells and induction of inflammatory responses in the graft. Indeed, we demonstrated within the liver itself significant upregulation of multiple genes involved in the detection, signaling, and execution of antiviral immune responses. Importantly, we found a marked increase in *CD8* gene expression. This finding is compatible with previous observations by Strenger et al [24] and Barozzi et al [25], who showed that iciHHV-6–positive individuals exhibit significantly heightened cytotoxic T-cell reactivity toward HHV-6 antigens (U54 or U90) in comparison to HHV-6–seropositive controls (without inherited chromosomal integration) [24, 25]. This is suggestive of chronic expression and priming of viral antigens encoded by the integrated HHV-6 genome.

The present work highlights the importance of pretransplant screening for iciHHV-6 and of monitoring reactivation to guide clinical decision-making. Our patient underwent a second successful LTx with modified induction and maintenance immunosuppression. Immediately after the second LTx, the patient also received prophylactic antiviral medication to prevent a potential reactivation of iciHHV-6B.

Particularly in the context of iciHHV-6, in which the baseline copies are conspicuously high, it is imperative to discriminate between latent and active infections. Such characterization can be substantiated with RNA- or antigen-based in situ analysis, in addition to nucleic acid quantification. In this work, we also introduced the sequence analysis of the viral intrahost genetic diversity as indicator of active replication [26]. Furthermore, our unique approach integrating the analysis of viral and mitochondrial DNAs showcases the relevance of comprehensive genomic analysis in unraveling intricate clinical traits.

Together, our findings contribute to the growing body of evidence indicating that iciHHV-6 can reactivate [22, 23, 27] and contribute to adverse clinical outcomes, particularly among immunosuppressed individuals [23].

Our work emphasizes the need for a deeper understanding of the impact of the virome in transplantation, bringing into focus the viruses persisting in a recipient, a chapter thus far overlooked. In this regard, we recently documented the coexistence of multiple DNA viruses, in unprecedented prevalences throughout the human body [11]. This viral flora residing within our tissues, the eukaryotic DNA virome, likely contributes significantly to the antigenic diversity between individuals, and must be comprehensively evaluated to assert its significance in immunosuppression and donor/recipient compatibility. Despite the absence of a clinical contextualization, a recent work by Lareau et al [28] demonstrated the existence of rare populations of HHV-6 super-expressors within allogenic chimeric antigen receptor T-cell products, and likely among the recipients’ endogenous CD4^+^ T cells. These subsets have the potential to contribute not only to the expansion of the viral reservoir but, most importantly, to the outcome of transplantation.

## Supplementary Data

Supplementary materials are available at *The Journal of Infectious Diseases* online (http://jid.oxfordjournals.org/). Supplementary materials consist of data provided by the author that are published to benefit the reader. The posted materials are not copyedited. The contents of all supplementary data are the sole responsibility of the authors. Questions or messages regarding errors should be addressed to the author.

## Supplementary Material

jiae268_Supplementary_Data

## Notes

***Author contributions.*** Conceptualization: K. H., T. J., and M. F. P. Methodology: L. H., L. P., D. P., J. L., S. S., and S. R.-D. Investigation: L. H., L. P., D. P., J. L., S. S., S. R.-D., and M. M. Visualization: L. H., L. P., S. S., and S. R.-D. Supervision: M. F. P. Writing—original draft: L. H., T. J., and M. F. P. Writing—review & editing: All authors.

***Acknowledgments.*** We acknowledge the Genome Biology Unit supported by HiLIFE and the Faculty of Medicine, University of Helsinki, and Biocenter Finland for the RNAscope imaging. The authors thank the IT Center for Science for supporting this project with computational and data storage resources.

***Disclaimer.*** None of the funding bodies had an input on the design of this study nor on the collection, analysis, or interpretation of data.

***Data availability.*** The complete consensus sequences of iciHHV-6 and mitochondrial DNA reconstructed from the explant, as well as the full mitochondrial genome of the implant, are available at GenBank with accession numbers OR643889, OR643891, and OR643892, respectively. The TRACESPipe [15] code is available at Zenodo (doi:10.5281/zenodo.7646369).

***Financial support*.** This research was funded by the Suomen Lääketieteen Säätiö (to L.P and M. F. P.); Medicinska Understödsföreningen Liv & Hälsa (to M. F. P.); the Valtion tutkimusrahoitus (to K. H.); Finska Läkaresällskapet (to K. H. and M. F. P.); Magnus Ehrnrooth Foundation (to K. H.); Jane and Aatos Erkko Foundation (to K. H.); the Rīgas Stradiņa Universitāte Research Grant (number 6-ZD-22/4/2023 to S. S., S. R.-D., and M. M.); and the Fundação para a Ciência e a Tecnologia, I.P., under the Scientific Employment Stimulus—Institutional Call (reference CEECINST/00026/2018 and project UIDB/00127/2020 to D. P.).
