# Supplementary material for: Reactivation of a Transplant Recipient's Inherited Human Herpesvirus 6 and Implications to the Graft
Source: J Infect Dis. 2024 May 20;231(2):e267–76. doi: 10.1093/infdis/jiae268 (PMC11841639; doi:10.1093/infdis/jiae268)
Supplement: jiae268_Supplementary_Data [file jiae268_supplementary_data.zip › Hannolainen_et_al_Supplementary materials_5.4.24.docx]

**Supplementary materials**

**Table of contents**

# Table of Contents

[Table of Contents 1](#_Toc144986029)

[Methods 2](#_Toc144986030)

[NGS](#_Toc144986031) and Data analysis

[Supplementary Tables 3](#_Toc144986036)

Suppl. Table 1. Viral DNAs detected in the liver biopsies by NGS and qPCR………………..….……………….3

[Supplementary Figures ….4](#_Toc144986037)

Suppl. Figure 1. Intra-host diversity of human herpesvirus 6B in the liver …………………………..………....4

Suppl. Figure 2. Electrophoretic visualization of amplification products by RT PCR………………………….5

Suppl. Figure 3. Detection of HHV-6 messenger RNA by in liver biopsies…………………………………………5

Suppl. Figure 4. Differential gene expression by whole transcriptome sequencing………………………….6

**Methods**

**NGS and data analysis**

Each sample was individually enriched via two rounds of hybridization, following the manufacturer's recommendations for low-input DNA (MyBaits v5 kit; Arbor Biosciences). The probes were 100 bp in length and designed with 2X tiling. Kapa Universal Blockers (Roche) were used to block unspecific binding to the adapters during hybridization.

During library preparation and viral enrichment, the libraries were amplified 3x13-25 cycles. The clean-up steps were performed with 1x KAPA HyperPure Beads (Roche). The enriched libraries were quantified with the KAPA Library Quantification Kit (Roche) using Stratagene 3005P qPCR System (Agilent) and pooled for sequencing on NovaSeq 6000 (one lane, S4, PE151 kit; Illumina).

Paired-end reads underwent trimming and merging with AdapterRemoval, eliminating ambiguous bases at the 5' and 3' termini that had quality scores less than or equal to two. Reads shorter than 20 bases were excluded from the analysis. FALCON-meta[16] was employed to identify the closest matching reference from the NCBI viral database. BWA[17] was utilized for aligning the reads with a seed length of 1000 and a maximum difference threshold of 0.01. Duplicate reads were removed with SAMtools[18], and the consensus sequences were reconstructed with BCFtools[19]. The coverage profiles were generated using BEDtools.

In cases of low breadth coverage (less than 15%), individual reads were manually inspected and verified through BLAST.

**Supplementary Tables**

|  | Timepoint  (month) | TTV | EBV | HCMV | HHV7 | B19V |
| --- | --- | --- | --- | --- | --- | --- |
| Explant | 0 | 6,15E+04 |  |  |  |  |
| Graft | 0 | 2,91E+02 |  |  | 1,29E+01 |  |
|  | 1 | 5,09E+06 |  |  |  |  |
|  | 2 | 9,80E+06 |  |  |  | 6,01E+03 |
|  | 3 | 8,96E+06 |  |  |  |  |
|  | 6 | 9,95E+06 | 2,07E+02 | 6,46E+00 | 1,94E+02 | 1,38E+03 |

**Supplementary Table 1.** **Viral DNAs detected in the liver biopsies by NGS and qPCR.** Prevalence of viral DNAs other than HHV-6B determined by NGS (grey boxes if positive) and qPCR (copy numbers/million cells if positive). The month 6 biopsy was not tested by NGS.

Abbreviations: TTV - Torque teno virus; EBV – Epstein-Barr virus; HCMV – human cytomegalovirus; HHV-7 – human herpesvirus 7; B19V - parvovirus B19.

**Supplementary Figures**

**
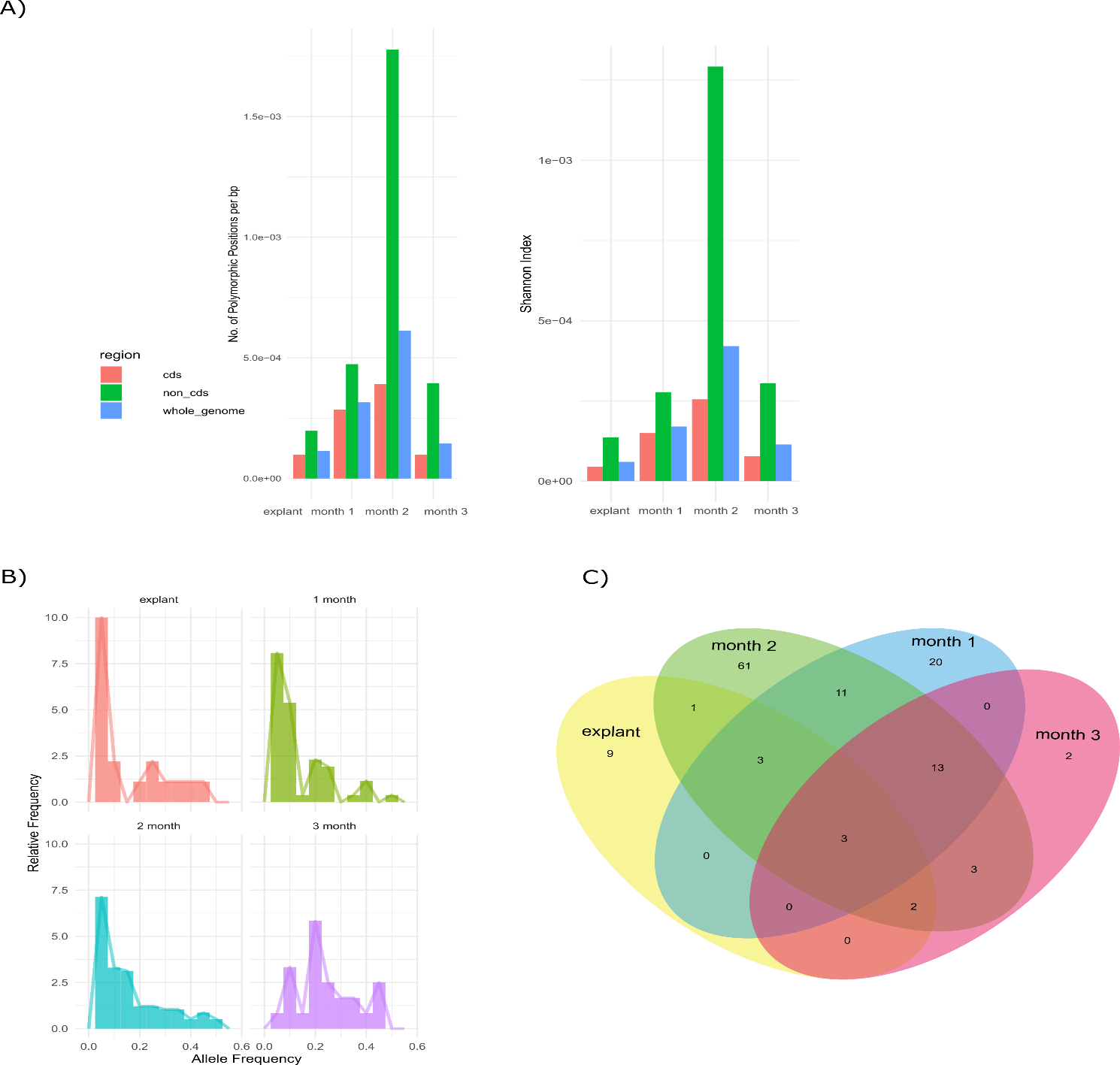
**

**Supplementary Figure 1. Intra-host diversity of human herpesvirus 6B in the liver.** (**A**) The genetic diversity of iciHHV-6B represented by the number of minor variant positions per base pair (left) and the per site Shannon index (right). This diversity is visualized across the entire genome, specifying coding sequences (CDS), and non-coding sequences (non-CDS) for different time points. (**B**) Site frequency spectrum displaying the distribution of minor variant allele frequencies across the different time points. (**C**) Venn diagram illustrating the number of minor variant positions that are unique to specific time points and those shared among samples.

**
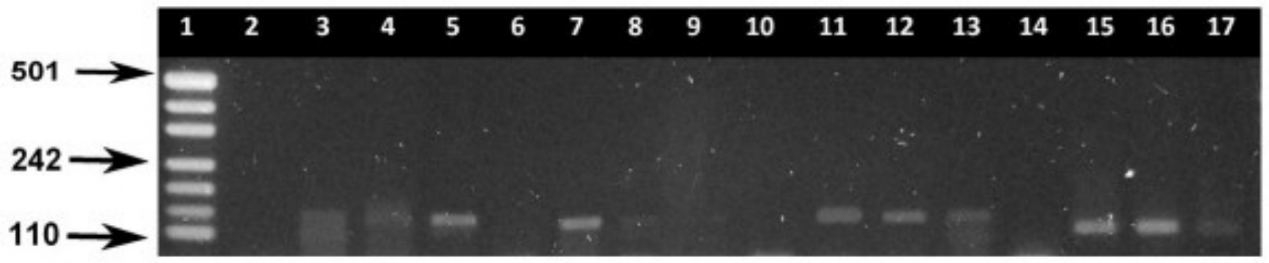
**

**Supplementary Figure 2.** **Electrophoretic visualization of amplification products by RT PCR**. Presented are the amplification products of human herpesvirus-6A/B U89/90 gene in a 2.5% agarose gel after reverse transcription polymerase chain reaction. **1**. pUC19 DNA/MspI (HpaII) marker; **2, 6, 10, 14** negative controls (molecular biology grade H2O); **3 – 5** implant at baseline; **7 – 9** implant at 6 months; **11-13** explant; **15 – 17** positive control.


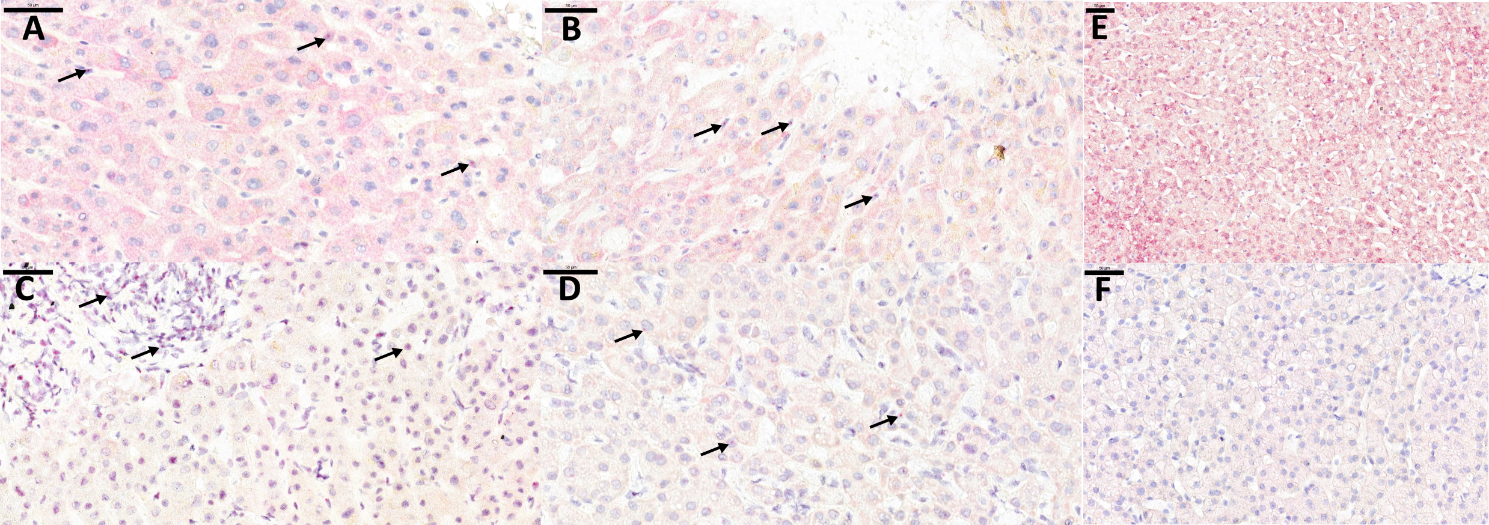


**Supplementary Figure 3.** **Detection of HHV-6 messenger RNA in liver biopsies**. Transcripts of the U94 late gene detected in the implant at (**A**) 1-, (**B**) 2-, (**C**) 3- and (**D**) 6 months post-transplantation and counterstained with hematoxylin. Positive and negative technical controls of (**E**) human PPIB mRNA probe and (**F**) bacterial DapB probe, respectively, were included. Representative positive probe signals are indicated with arrows. Scale bar of 50 μm in the left upper corner. Images captured with CaseViewer 2.4

**
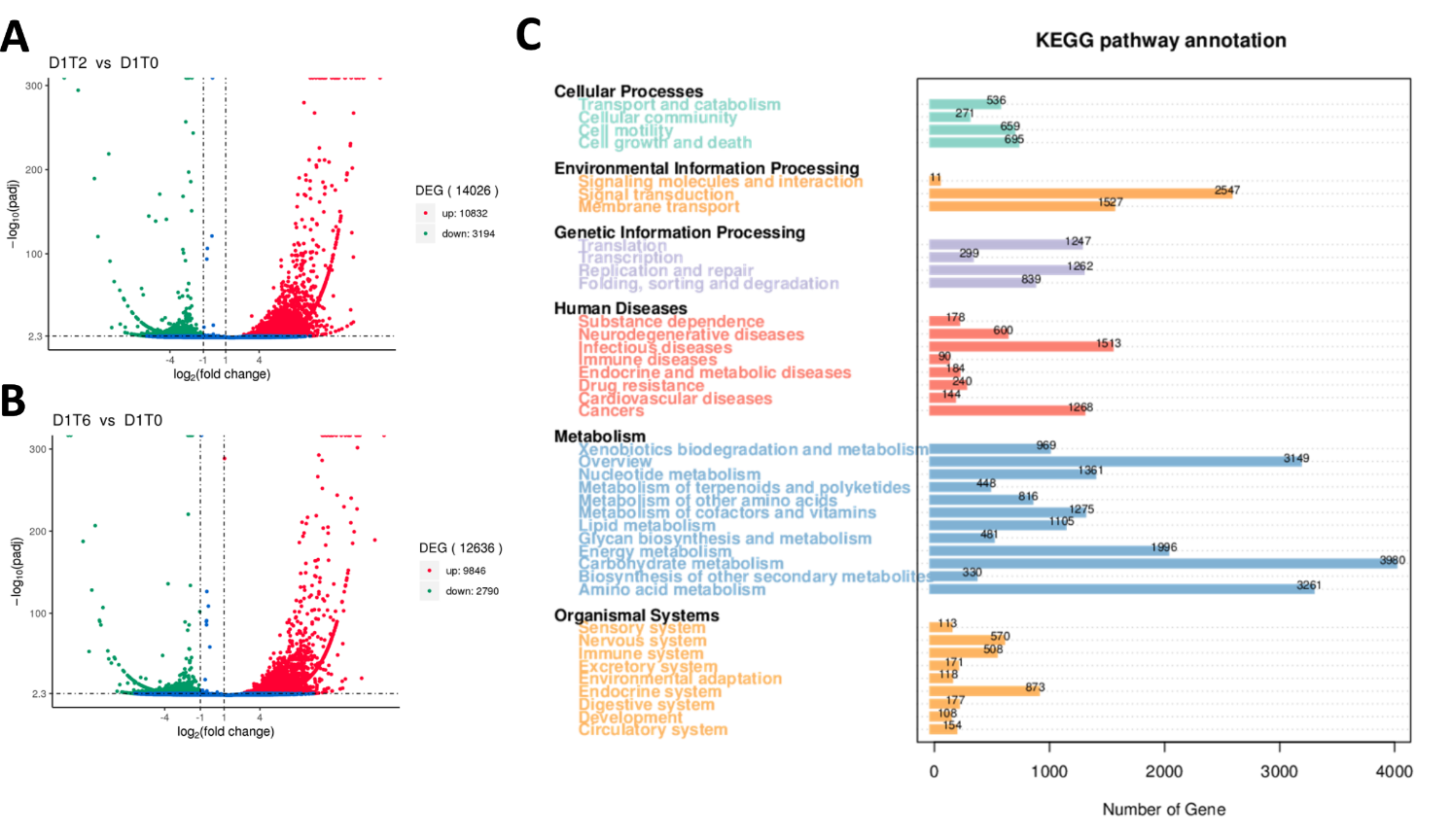
**

**Supplementary Figure 4. Differential gene expression by whole transcriptome sequencing.** Volcano plots representing differentially expressed genes between (**A**) baseline and 2 months post- LTx and (**B**) baseline and 6 months post LTx. Represented in (**C**) is the KEGG annotation of differentially expressed pathways.
