# Supplementary figures and images for: Reactivation of a Transplant Recipient's Inherited Human Herpesvirus 6 and Implications to the Graft
Source: J Infect Dis. 2024 May 20;231(2):e267–76. doi: 10.1093/infdis/jiae268 (PMC11841639; doi:10.1093/infdis/jiae268)

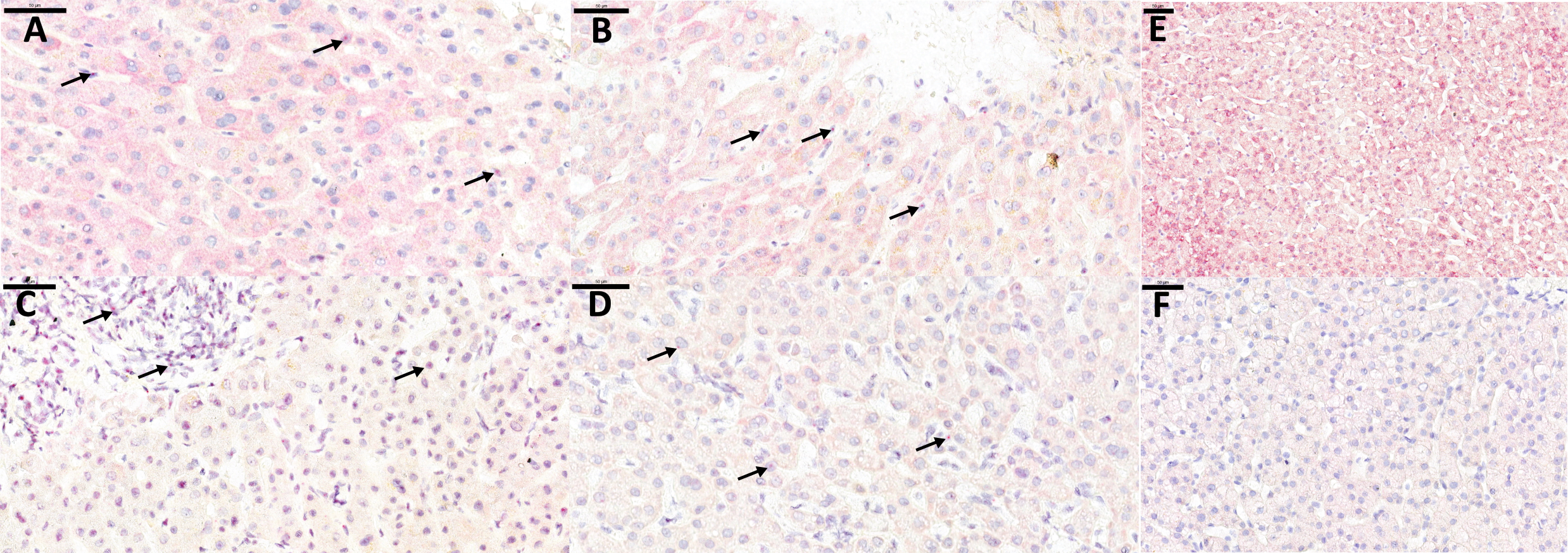

Supplement: jiae268_Supplementary_Data [file jiae268_supplementary_data.zip › Supplementary Figure 3.tiff]

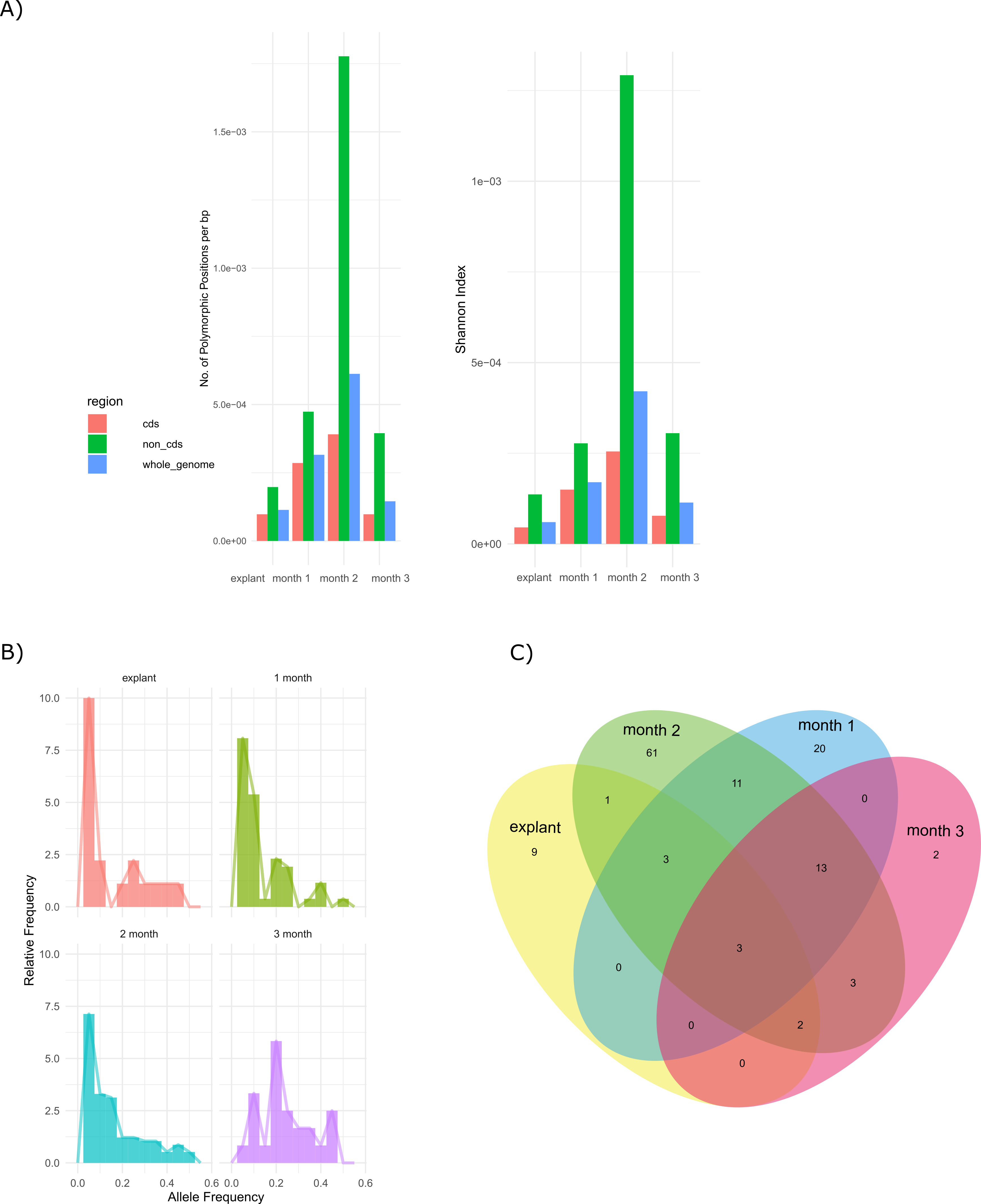

Supplement: jiae268_Supplementary_Data [file jiae268_supplementary_data.zip › Supplementary Figure 1.tiff]

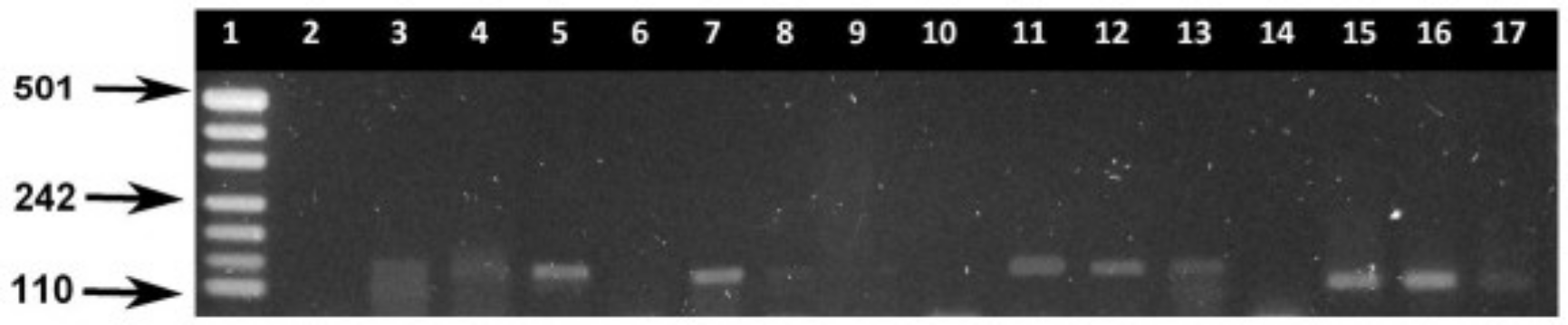

Supplement: jiae268_Supplementary_Data [file jiae268_supplementary_data.zip › Supplementary Figure 2.tiff]

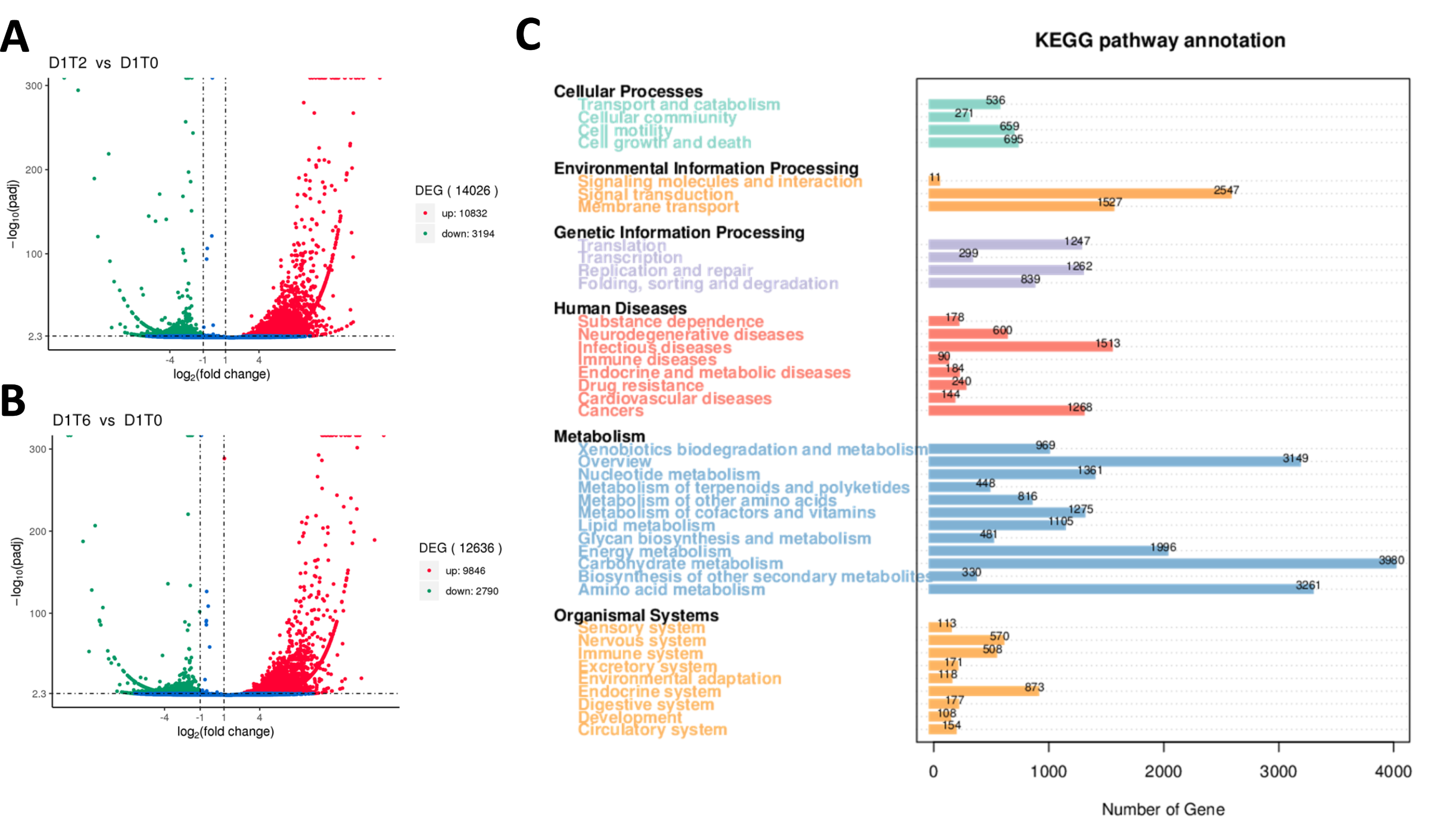

Supplement: jiae268_Supplementary_Data [file jiae268_supplementary_data.zip › Supplementary Figure 4.tiff]
